# Sport Practitioners as Sport Ecology Designers: How Ecological Dynamics Has Progressively Changed Perceptions of Skill “Acquisition” in the Sporting Habitat

**DOI:** 10.3389/fpsyg.2020.00654

**Published:** 2020-04-24

**Authors:** Carl T. Woods, Ian McKeown, Martyn Rothwell, Duarte Araújo, Sam Robertson, Keith Davids

**Affiliations:** ^1^Institute for Health and Sport, Victoria University, Melbourne, VIC, Australia; ^2^Port Adelaide Football Club, Football Department, Adelaide, SA, Australia; ^3^Department of Sport and Exercise Science, James Cook University, Townsville, QLD, Australia; ^4^Centre for Sport and Human Performance, Sheffield Hallam University, Sheffield, United Kingdom; ^5^CIPER, Faculdade de Motricidade Humana, University de Lisboa, Lisbon, Portugal

**Keywords:** constraints-led approach, ecological dynamics, self-learning and preparation for performance, practice designs, skill adaptability

## Abstract

Over two decades ago, Davids et al. (1994) and Handford et al. (1997) raised theoretical concerns associated with traditional, reductionist, and mechanistic perspectives of movement coordination and skill acquisition for sport scientists interested in practical applications for training designs. These seminal papers advocated an emerging consciousness grounded in an ecological approach, signaling the need for sports practitioners to appreciate the constraints-led, deeply entangled, and non-linear reciprocity between the organism (performer), task, and environment subsystems. Over two decades later, the areas of skill acquisition, practice and training design, performance analysis and preparation, and talent development in sport science have never been so vibrant in terms of theoretical modeling, knowledge generation and innovation, and technological deployment. Viewed at an ecological level of analysis, the work of sports practitioners has progressively transitioned toward the facilitation of an evolving relationship between an organism (athlete and team) and its environment (sports competition). This commentary sets out to explore how these original ideas from Davids et al. (1994) and Handford et al. (1997) have been advanced through the theoretical lens of ecological dynamics. Concurrently, we provide case study exemplars, from applied practice in high-performance sports organizations, to illustrate how these contemporary perspectives are shaping the work of sports practitioners (sport ecology designers) in practice and in performance preparation.

## Introduction

The gardener cannot actually “grow” tomatoes, squash, or beans – she can only foster an environment in which the plants do so.

– Stanley McChrystal

This is an exciting era for sports practitioners and applied scientists interested in understanding how to help athletes “grow and flourish” in complex performance surroundings. In a high-performance sport environment, the significant aims of coaches, sport scientists, and performance analysts are to develop “athletes of the future” and prepare “athletes of the present” for competitive performance. To foster successful interactions of athletes and teams with competitive performance and practice environments, the areas of skill acquisition, practice and training design, performance analysis, and talent development have never been so vibrant in terms of theoretical modeling, knowledge generation, technological deployment, and the application of innovative ideas in practice, training, and performance preparation. Viewed at an ecological level of analysis, the work of sports practitioners is to facilitate a productive, evolving relationship between an organism (athlete and team) and its environment (sports competition).

Indeed these ideas were originally promoted in sport science over two decades ago in theoretical concerns raised with traditional, mechanistic perspectives of movement coordination (Davids et al., 1994) and skill acquisition (Handford et al., 1997). Those position papers advocated the potential of an ecological approach to sport scientists and scrutinized reductionist information-processing perspectives on human performance dominant at that time. An important insight was that skill “acquisition” was conceptualized to emerge from an *evolving practice ecology*, which necessitated sports practitioners to appreciate the complex, deeply integrated, and non-linear reciprocity of the organism (performer), task, and environment subsystems (Newell, 1986). Such a theoretical conceptualization challenged the traditional perspectives of skill acquisition, having profound implications for understanding the performer–environment relationship and for how sports practitioners viewed their role in the preparation of athletes for performance. Here we seek to examine the progress made on complementing that emergent consciousness through the contemporary theoretical lens of ecological dynamics, exploring how the original ideas have been advanced in the intervening decades. We also examine case studies showing how the key concepts are currently shaping the work of some sports practitioners in practice and in performance preparation.

An ecological dynamics rationale, integrating ecological psychology, dynamical systems theory, the complexity sciences, and evolutionary science, views skilled behavior as the emergence of functionally adaptable performance solutions (i.e., actions, for a detailed review, see Araújo et al., 2020). In this framework, behavior is a self-organizing phenomenon that emerges from the continuously dynamic interplay of an organism’s characteristics and the *affordances* (possibilities for action: Gibson, 1979) offered in a specific competitive performance environment (Araújo et al., 2006). Thus, skilled behavior evolves over timescales of performance, learning, and development (Button et al., 2020). These theoretical propositions are grounded in James Gibson’s (1979) theory of direct perception in ecological psychology and in Scott Kelso’s seminal work on coordination dynamics (e.g., Kelso, 1981a, b, 1984). Specifically, Gibson (1979) proposed how detection of information regulated action (and *vice versa*) and how the realization of affordances underpinned functional behaviors in dynamic performance environments. In a series of laboratory experiments, Kelso observed inherent, spontaneous self-organization tendencies in human movement systems and sudden phase transitions between states of coordination as the participants interacted with informational constraints of the environment (Kelso, 1981b, 1984).

In this commentary, we discuss how the role of a sports practitioner has shifted through the application in sport science of these key ideas in ecological psychology, behavioral neuroscience, and human movement science. Sports practitioners have moved on from an instrumental role of ensuring compliance of performers with “operational standards” or “technical performance templates” defined in coaching and performance manuals toward the designer of a learning ecosystem, working in multidisciplinary teams, to promote emergent, self-organized athlete–environment interactions. We highlight how this role perspective focuses more attention on the adaptability of athletes in performance, predicated on being excellent learners. The aims of this commentary are to: (1) provide an appreciation of advances in key concepts in ecological dynamics made in the past two decades and (2) provide (brief) practical insights from case studies in high-performance sport describing how this ongoing conceptualization is facilitating the implementation of practice designs inviting effective behaviors.

## Part 1: Skill Acquisition as an Evolving Practice Ecology – an Update

### A Progression Toward Ecological Dynamics

A critical theoretical tenet of the ecological approach to skill acquisition, highlighted by Handford et al. (1997), is the appreciation of the performer–environment mutuality. From an ecological perspective, the “environment” refers to an animal’s surroundings within which it can perceive and act, changing the environment and their deeply entwined relationship with it (Gibson, 1979). These relationships can be changed across different timescales (in sport, evolving along the macro-scale of talent development and changing within the micro-structure of practice; see Davids et al., 2017; Balagué et al., 2019). Thus, actions and behaviors should be understood as the result of specialized relationships that emerge between an organism and its environment (Handford et al., 1997). More directly, behaviors and actions do not appear in a vacuum. An athlete’s behaviors cannot be understood without sustained reference to the specific environmental context in which they emerge (Renshaw et al., 2009). Specifically, the ecological dynamics approach focuses less on the putative control mechanisms of organisms, like internalized representations and knowledge structures stored in memory, and more on the reciprocal nature of perception and action which supports performance functionality. This was captured elegantly by Beek and Meijer (1988, p. 160) as the appreciation of “phenomena within the organism–environment synergy rather than within the organism *per se*.” This more biophysically oriented theoretical conceptualization subsequently rejects the more mechanistic traditions of mental information-processing theories of skill acquisition. Such theories historically view movements as idealized, internalized templates for actions that originate from the mind and which are optimized with practice, rather like a computer programmer “debugs” a piece of software (for an original overview of implications for sports science; see Davids et al., 1994).

### Organismic Asymmetry in Human Behavior

This inordinate emphasis on internalized representations somehow acquired in the mind of the athlete is another example in science of a dualism, in this case mind–body, proposed in explanation of natural physical phenomena (Turvey and Shaw, 1995). A prominent example is the confected “nature vs. nurture debate” to discuss exclusive influences on human behaviors such as learning, intelligence, propensity to disease, and expertise. The manifestation of this organism–environment dualism was recognized by Dunwoody (2007) who criticized the inherent bias caused by “organismic asymmetry” in the study of human behavior. Dunwoody (2007) identified one such organismic asymmetry as neglecting the foundational *person–environment relationship* as an interrelated basis for explaining human behavior, in favor of a biased preference for organismic-centered mechanisms such as internal mental models of the world. Brunswik (1955) indicated that, in organism–environment interactions, it was considered that both equally contribute to the organization of behavior. Brunswik (1955) noted a bias in most psychologists for attributing achievement to the internal process of humans, rather neglecting the influence of the environment in co-shaping human behaviors. Typically, much cognitive psychology remains focused on conscious mental life, with little reference to the role of the environment in shaping behavior (Davids and Araújo, 2010).

In 2011, Araújo and Davids highlighted the relevance of organismic asymmetry to sport scientists seeking to understand how athletes self-organized during practice and performance. This theoretical re-positioning offered significant implications for how sports practitioners could learn to rely less on traditional approaches to athlete development and preparation for performance, which emphasized verbal instructions and corrections, constant repetitions to “optimize a movement pattern,” and the internalization of rehearsed behavioral reactions and responses in training. Indeed this theoretical re-positioning was in agreement with the empirical work conducted by Schöllhorn and colleagues, who demonstrated both inter-individual (Schöllhorn and Bauer, 1998) and intra-individual (Schöllhorn et al., 2002) variability and differences with regards to movement patterning, highlighting the fallibility of sport pedagogies grounded in the (attempted) acquisition and reproduction of “optimal” movement patterns. To further exemplify, an organismic asymmetry can be detected in some current notions of the concept of self-regulation in human behavior. Traditionally, self-regulation has been defined from a cognitive orientation referring to all the “self-generated thoughts, feelings, and actions that are planned and cyclically adapted to the attainment of personal goals (Zimmerman, 2000, p. 14). The bias toward the internalized regulation of behavior through planned goal achievement is apparent. From an ecological dynamics rationale, self-regulation can be conceptualized in a broader behavioral framework, emphasizing an individual’s emergent interactions with the environment rather than referring to behaviors that are guided by internalized plans and goals with little reference to environmental interactions. In ecological dynamics, individuals can learn to self-regulate by developing and exploiting a deeply intertwined relationship between their actions, perceptions, intentions, and emotions to continuously support these emergent interactions. By harnessing this functional relationship with a performance environment, athletes learn to self-regulate by adapting stable action–perception couplings developed in rich and varied practice environments.

### Variability and Performance

In their position statement, Handford et al. (1997) suggested that there was an over-emphasis on the measures of performance outcome variability (such as standard deviations and coefficients of variation) in sport and movement science research, which was focused on the magnitude of variability in task outcomes. This is only part of the picture and biased to the view that variability was often equated with “noise” or error in humans, considered as information-processing channels. This conceptualization was due to the linear movement models that were popular in motor behavior theories in the 1960s to the 1970s and that somewhat still prevail in current practice. Contemporary models of movement, such as ecological dynamics, advocate that humans and groups are *complex adaptive systems* with inherent non-linear properties. Variability in such systems needs to be much more carefully interpreted in a nuanced way, which is the challenge for sports practitioners interested in enhancing athlete and team performance.

Complex systems with many degrees of freedom can be seen as a “curse” (of organization, coordination, and control) or “blessing” (adaptability, re-organization, and functionality) as was discussed by Handford et al. (1997). The blessing is that athletes can continuously be encouraged to exploit self-organizing tendencies in their movement systems to form synergies (coordination patterns). This is where the variability can be functional. However, it is important to recognize that variability in movement patterns can be detrimental. Variability does not just exist within coordination and can manifest at different levels within an individual’s kinematic profile. One level consists of fluctuations in individual elements such as joints and segments, usually seen in novices and considered less than desirable. Another perhaps is whole system variability, where several coordinated elements combine to produce an overall movement pattern, which manifests itself in *system degeneracy* (Edelman and Gally, 2001). At the first level, there is consistent evidence that variability decreases as skill level increases (Button et al., 2003; Bradshaw et al., 2009; Fleisig et al., 2009; Betzler et al., 2012; Hiley et al., 2013). It could be hypothesized that higher levels of movement variability in the lower skilled athletes at this level are reflective of them searching for effective movement patterns in line with the degrees of freedom and U-shaped curve hypotheses. However, the consistent decrease in individual variability as skill levels increase may be evidence of the need to constrain element variability to facilitate functional coordination and allow multi-element coordination variability to emerge (Button et al., 2020). Understanding the change in variability profile at each level, in particular during any interaction with motor learning and/or adaptation, could provide insight into how any functional role of variability emerges.

One of the functions attributed to movement variability is facilitation of the adaptation of an organism to changing environmental and task constraints (Davids et al., 2003; Glazier and Davids, 2009). In discrete movements, this type of variability is different to the undesired variability in the endpoint or the outcome of the movement (e.g., the number of targets accurately hit). Functional movement variability is now considered to be a characteristic of highly skilled movers (Button et al., 2003; Wilson et al., 2008), and an individual’s variability profile is thought to change during task learning. For example, a U-shaped curve has been hypothesized to characterize coordination variability across skills, where the highest and lowest skilled display increased variability while those in intermediate stages have their variance constrained (Wilson et al., 2008).

In summary, the challenge for sports practitioners is to sort what is “good” (functional) variability from “bad” (dysfunctional) variability in an individual athlete’s performance [see Scholz and Schöner (1999) and Latash et al. (2010) on the Uncontrolled Manifold Hypothesis]. At this stage, it is worth drawing attention to the influential theoretical insights and experimental data of Bernstein’s (1967) which highlighted the need for psychologists, movement scientists, and sport scientists to re-consider how measures of movement variability should be conceptualized for human performance. Movement pattern variability can support the skill adaptations needed as the influence of task constraints on athlete behaviors emerges during practice and performance. The implications for practice and performance were captured in the phrase of “repetition without repetition,” indicating how practice designs for trainers and coaches should provide opportunities for athletes to solve performance problems in different ways using a variety of behaviors.

### Skill Adaptation

This re-conceptualization of self-regulation and functional variability has important implications for the translation into practice in sports performance preparation, suggesting that the commonly used term skill “acquisition” does not actually involve the *acquisition* of a physically reproducible motor memory stored in the brain. Rather, a more relevant description of the learning process in sport may be considered as “skill adaptation” (Araújo and Davids, 2011). What is developed is a highly functional relationship that evolves between an athlete and a competitive performance environment over extended timescales: a flourishing relationship that is supported by learning, experience, growth, and development (Seifert et al., 2013). Interestingly, this conceptualization of skill acquisition, predicated on continuously growing athlete functionality, was foreshadowed by Bernstein’s (1967, p. 134) notion of *dexterity*, which he defined as the *“the ability to find a motor solution for any external situation, that is, to adequately solve any emerging motor problem correctly* (i.e., adequately and accurately), *quickly* (with respect to both decision making and achieving a correct result), *rationally* (i.e., expediently and economically), *and resourcefully* (i.e., quick-wittedly and initiatively)” (italics in the original). Furthermore, according to Bernstein, the “demand for dexterity is not in the movements themselves but in (adapting to) the surrounding conditions” (Bernstein, 1996, p. 23). In this respect, Bernstein’s’s (1967) insights foreshadowed how dexterity could provide a foundation for skill adaptation, with his definition of dexterous behavior showing the deeply intertwined links between cognition, action, and perception, the interaction of which is continually used to negotiate a dynamic performance environment. His ideas clarified how movement variability and skill adaptation are founded on the self-organization tendencies that can be exploited in dynamic performance contexts (Chow et al., 2011).

These theoretical insights on athlete performance illustrate the fundamental importance of many natural phenomena in the environments studied by ecologists, exemplified by the inherent self-organizing tendencies observed in complex systems formed by shoaling fish, flocking birds, synchronization of insect emission of sound and light as information, and the exploration of growing conditions by plants or mosses (Passos et al., 2013). Self-organization tendencies are ubiquitous in nature. Based on the key principle of “information–action coupling,” these tendencies have even been observed in single-cell organisms without a nervous system (Boisseau et al., 2016). The dynamics of self-organization have drawn attention to the fundamentality of the organism–environment relationship, predicated on actions regulated by surrounding information, emphasizing the ecological systems at the heart of these links. It is important to note that the self-organizing tendencies in ecology are rarely expressed in isolation of a context (i.e., what is happening in the environment). For example, the organizing principle in a self-organizing system like a shoal, with each fish functionally co-adapting with each other, concerns their emergent co-movements (remaining within one “fish” length of each other) relative to those of an approaching predator or food source (informational constraints). The emergence of these rich and sophisticated global behavioral patterns in complex neurobiological systems is not pre-programmed within a knowledge structure shared between each single fish in the shoal nor pre-orchestrated by a piscatorial “leader” (acting as a collective system “coach”). Rather, they emerge from the information created by the movements of each complex system component, continually co-adapting to each other.

### Implications of These Ideas for Sports Practitioners: Representative Design

Through an ecologist’s perspective, an important part of a sports practitioner’s role is to identify the critical informational sources or, more technically, the *affordances* (defined as opportunities or invitations for action; Gibson, 1979; Withagen et al., 2012) of a training setting that are likely to impact an athlete’s or a teams’ behaviors (similar to an ecologist being cognizant of how the presence of a predator or food source shapes the time–space relations underlying the emergent patterns of behavior of each fish within the shoal as a collective). Understanding the relevant affordances used to regulate performance behaviors allows groups of practitioners to carefully coordinate the design of learning activities that *represent*, or closely simulate, the demands of competitive performance contexts. While Handford et al. (1997) addressed the issue of specificity of practice, later work in ecological dynamics precisely located the key issues for sport practitioners as ensuring *representative design* after insights of Brunswik (1955) (see Araújo et al., 2005, 2006, 2007). The ensuing work of Pinder et al. (2011a, b) drew the attention of sport scientists and sport practitioners to the relevance of this concept for ensuring that the task constraints of learning sessions, especially informational constraints, represented (that is faithfully simulated) those of competitive performance environments. It is through the prolonged exposure to representative practice tasks that a performer learns to attune to (or “detect”) the information sources that specify the relevant properties of the affordances of their environment using a variety of modalities such as haptic, visual, and auditory sensory systems [i.e., a surfer progressively learning to detect the motion of a wave (using haptic and visual sensory systems) to inform a “cutting” manoeuver used to score points in competition] (Withagen et al., 2017). The ongoing process of attunement to performance opportunities helps athletes and teams to develop a more functional and adaptable relationship with a particular competitive environment. More specifically, if we consider a performance environment as a *rich landscape of affordances* (Rietveld and Kiverstein, 2014), some of them designed by the coach when presenting practice tasks, then such practice tasks are directing or guiding the search of the performers. Moreover, some affordances can attract or invite the athletes to act upon them, especially if they precisely match the current capacities, abilities, and skills [termed “effectivities” by Gibson (1979)] of the athlete and the task constraints channel the athlete toward them (Araújo et al., 2019). From this perspective, affordances have both body-scaled (e.g., limb lengths) and action-scaled (e.g., strength output) properties that are perceived relative to the performer’s current action capabilities (Fajen et al., 2009). This idea is most important to consider in athlete development programs in high-performance sport.

The current thinking on the affordance landscape notion for practice design suggests that, with experience, skill, and quality of practitioner support, athletes can become increasingly competent at perceiving and utilizing the most *soliciting* of affordances. This process is predicated on strong coupling tendencies between the presented affordance landscape and the skill of the athletes’ perception and action in specific environmental designs (Withagen et al., 2017). Thus, through the landscape design, the practitioner can “nudge” or guide the athlete to use specific affordances while ignoring other less relevant ones. This ecologist’s perspective leads to another important tenet of ecological dynamics for sports practitioners, that of *synergy formation* and *self-organi*zation *under constraints*.

### Synergy Formation in Athletes and Sports Teams Exploits Self-Organization

To assist with the understanding and subsequent explanation of synergy formation, it is important to, firstly, appreciate the theoretical roots of ecological dynamics. Ecological dynamics is grounded in theoretical approaches, such as direct perception in ecological psychology, explaining how (detection of) information regulates actions and actions are coupled to perception of affordances (Gibson, 1979). At its core, it provides scientists with a framework for describing the emergence of complex, non-linear, and self-organized behaviors shaped by task, organismic and environmental constraints (Newell, 1986), and the order parameter–control parameter relations underpinning the dynamics of coordination in nature (Kelso, 1981a, b, 1984). Newell (1986) modeled how nested, interacting task and organismic and environmental constraints shaped coordination development, later applied to coordination behaviors and their acquisition in sport performance (Davids et al., 1994; Handford et al., 1997; Renshaw and Davids, 2004). Kelso (1981a, b, 1984, 1995) produced data showing how the coordination dynamics of brain and behavior shaped perceptions, intentions, and actions, during performance and learning, not as separated entities stored in the brain but as self-organizing patterns of behavior formed through the interaction of system components (order parameters) and the critical informational constraints of the environment (control parameters) (Kelso, 1995). In the central nervous system, the functioning of “system components” is observed at a macroscopic level, such as the stimulation of neurons simultaneously firing. In human movement, muscles of different limb segments synergistically interact to form multi-articular actions (Kelso, 1992, 1995). The interaction of system components with critical informational or environmental constraints results in the emergence of coordinated, self-organized behaviors (Kelso, 1981b; Kugler and Turvey, 1987). Ecological dynamics, therefore, fundamentally blends key concepts and insights specific to ecological psychology and dynamical systems theory in the explanation of synergy formation and coordination of action in complex neurobiological systems (for further insights, see Araújo et al., 2006; Warren, 2006).

The initial implications of these theoretical ideas for sport practitioners were raised by Handford et al. (1997) in a discussion of coordination and its acquisition. Gradually over the years, several lines of research began to reveal how these applied scientific insights had radical implications for the work of sport practitioners interested in how athletes coordinated their actions in sport collectives at a mesoscopic level, for example, in synchronized swimming and diving, cycling in a group, and especially in team sports (e.g., Passos et al., 2009; Duarte et al., 2012, 2013; Vilar et al., 2012; Silva et al., 2014; Passos and Davids, 2015; Ric et al., 2016). Over the following two decades, key insights on processes of co-adaptation were raised for understanding the functioning of 1v1 dyadic systems in team sports like basketball (Bourbousson et al., 2010a, b), association football, rugby union, and small sub-groups of athletes in sub-phases of play (e.g., 4v2 in rugby union, 6v6 in association football, 5v5 in futsal) (for empirical examples, see Araújo et al., 2006; Araujo et al., 2014; Passos et al., 2009). These insights now theoretically guide applied scientific work in the fields of performance analytics and biomechanics, sports pedagogy, tactical behaviors in team sports, physiology, skill acquisition, and practice design (Travassos et al., 2013; Araújo and Davids, 2016; Ribeiro et al., 2019a). From an ecological dynamics perspective, the processes of performance and functionality in sport can clearly draw inspiration from biological systems which function in a symbiotic way to flourish together in a specific environment. In sport and other ecological systems, function is predicated on information that reciprocally shapes the ongoing evolution of co-habiting organisms in a particular environment, with each organism shaping the environment while being shaped by its surrounds.

### The Coach as the “Designer”

One of the key issues raised by Handford et al. (1997) was that the learner needed to be placed at the center of the learning process, with less of an emphasis of the coach being at the center of the instructional process. Over the past two decades, the ecological dynamics framework has emphasized how the role of the sports practitioner has evolved from an autocratic instructor who leads every sequential step of athlete progression through continuous provision of verbal information and corrective feedback to one of a “learning designer” whose role it is to work with athletes to identify and manipulate the key constraints of practice environments (Davids, 2012, 2015). This co-designing learning activity places the athlete and his/her needs at the heart of the development and performance preparation process. This is likely to augment the design of representative practice tasks as the coach and the athlete work together to co-design critical affordances that the athlete attunes to, thus guiding their behaviors. Traditionally, for example, the role of the coach has been conceived in a hierarchical way, sometimes even autocratically, preparing athletes and teams for performance through a strong emphasis on *global-to-local* synergy formation processes to externally regulate dynamics in performance and learning (Ribeiro et al., 2019b). In team sports, this can be typically exemplified through an external agent (i.e., coach, instructor, and trainer) prescribing strategic patterns of behavior in specific phases of a game. Conversely, an ecological dynamics framework advocates *local-to-global* synergistic tendencies, in which a system’s synergy formation tendencies can be exploited in self-organization through interactions with the performance environment designed into representative practice tasks (Ribeiro et al., 2019a, b). Buekers et al. (2019) have re-iterated this point by arguing that the tactical performance of players in sports teams can be understood with respect to the ecological laws governing the perception of information in surrounding energy arrays during performance (aligned with the local-to-global synergy formation tendencies in sports teams). Team sports strategizing, on the other hand, is focused on the adherence to a performance plan prepared in advance (global-to-local synergy formation emphasized, often being led by a coach as Ribeiro et al., 2019b, noted). More recently, this distinction between different pedagogical approaches has been focused on the differences between the more traditional, command-driven practices of “hard education” and eliciting of learning opportunities in practices of “soft education” (van der Kamp et al., 2019).

So, How Does a Sports Practitioner Design a Learning Environment That Places the Athlete at Its Center and Appreciates the Bi-directional Nature of Synergy Formation to Enable the Rich Behavioral Patterns That Self-Organize at Both Intra-individual (Within an Athlete) and Inter-individual (Between Athletes) Levels?

In early recognition of the above question, Handford et al. (1997) paid particular attention to the manipulation of task constraints for sports practitioners, suggesting that it implied a more “hands-off” approach to sport pedagogy. Rather like an ecologist, the practitioner can create conditions for an athlete to exploit and flourish during the development and learning process. The implication is that a practitioner did not need to intervene and “nourish” an athlete continually but instead can work with the individual organism to adapt to the surrounding environment and flourish by getting everything needed from interactions with environmental constraints.

While this descriptor of hands-off coaching to prevent hyperactive verbal interference from coaches has been well understood and heeded over the past decades, there have been some indications that the new role, aligned with an ecologist, has been mis-conceptualized by some in a literal sense. To clarify, *hands-off coaching* signals a shift to a deep understanding of task, personal, and environmental constraints on individual learners and finding ways to co-design learning environments replete with affordances to guide each learner toward active exploration of a range of performance solutions. The role of practitioners, therefore, has become more important than ever, evolving from a prescriptive instructor with complete control over the whole process (hands-on) to a learning designer deeply integrated as a member of a team of sports practitioners focused on athlete performance and development at all stages. An important point to highlight in the hands-off approach is that, instead of offering their pre-programmed task solutions (according to the personal view of the coach), coaches need to work with the athlete to find individualized creative solutions for a performance problem. In this way, coaches are guiding the athletes to find solutions to the unknown problems that they may face in future competitions, not just repeating solutions for the training task problems (Araújo et al., 2009). For example, both tactical and strategical work in contemporary methods for preparation for team sports performance are now predicated on “Big Data” and technology implemented by teams of sports practitioners within the framework of an ecological dynamics rationale for learning designs in practice programs (Woods et al., 2019b; Browne et al., 2019).

### A Department of Methodology: A Platform for Integrative Sport Science and Coaching

Although a theoretical and applied move toward practitioners as learning designers is welcome, some practitioners may be locked into traditions of practice and performance that advocate deterministic models of human behavior (e.g., Chow and Knudson, 2011), leading to coach-centric and hands-on approaches (resulting in rather over-dominating performance preparation). Practitioners who are guided by historic traditions of supporting athlete performance and development (a type of “path dependency” or acculturation process) can be subjected to “system capture.” System capture occurs when the work of a sport practitioner is not guided by a theoretical framework of athlete development and performance but rather is captured by “operational standards” defined in coach education manuals that promote “optimal” performance templates (Rothwell et al., 2020). System capture of this nature can inhibit the development of innovative methods of athlete support and also disrupt multidisciplinary sport science teams when collaborating to design learning environments. The result is that practice and performance dissonance amongst practitioners could lead to “silo” working (Springham et al., 2018) and disjointed athlete preparation practices. One way for practitioners to avoid system capture and operate effectively as learning designers is to work collaboratively in a department of methodology (DoM) (Rothwell et al., 2020).

A DoM in an applied sport habitat should be composed of a group of practitioners and applied scientists who share integrative tendencies based on a rich mix of empirical and experiential knowledge. The aim of a DoM would be for group members to work within a unified theoretical framework (i.e., ecological dynamics) to: (i) coordinate activity through shared principles and language to avoid working in “silos,” (ii) provide an integrative platform to communicate coherent ideas, (iii) collaboratively design practice landscapes rich in information (i.e., visual, acoustic, proprioceptive and haptic), and (iv) guide the emergence of multi-dimensional behaviors in athlete performance (Chow et al., 2011). In addition, as foreshadowed by Davids et al. (1994) a DoM can support practitioners and applied scientists to bridge the gap between theory and practice to enable the design of highly integrated and representative learning tasks. Since Newell’s (1986) model focused on the integrated interacting constraints related to the individual, task, and environment, the nested relationship between them advocates the need for practitioners to collaborate together in a DoM to prevent sport practitioners from treating each constraint in isolation (Rothwell et al., 2020). As recently discussed by Woods et al. (2019b), the contemporary practice design of this nature requires an effective multidisciplinary approach, where a team of practitioners such as performance analysts, coaches, sport psychologists, sport scientists, and skill acquisition specialists, can work collaboratively in a DoM to analyze, sample, integrate, and manipulate nested practice task constraints on each individual athlete based on evidence from large sets of competitive performance data. This contemporary multidisciplinary approach would likely resolve behaviors that are perceived to be desirable for team and/or athlete success (product) in addition to the resolution of the interacting constraints that shape their emergence (process). Such information creates the basis for representative learning designs in practice and training. Further, this approach would likely lead to innovation in practice design as sport practitioners would not simply follow sequential steps advocated in coaching manuals as a result of path dependency. Rather, sports practitioners would identify critical sources of information within a competitive environment perceived to impact an individual athlete’s performance behaviors and create an ecosystem that augments an athlete’s perceptual attunement (i.e., detection) to relevant affordances in the landscape. In this respect, practitioners and applied sport scientists should focus the learning and practice design on a deeply intertwined relationship between value (affordances) and meaning (information) to support the development of highly attuned athletes. Affordances immediately (directly) indicate their value of use in an environment where structured patterns of (visual, acoustic, haptic, and proprioceptive) information (energy) reveal what objects and surfaces are (i.e., their meaning; Withagen et al., 2012). Accordingly, from an ecological dynamics’ perspective, an athlete would not “acquire” an idealized skill. Rather, over time, he/she would develop a deeply functional and adaptive relationship with the performance environment (Araújo and Davids, 2011).

In the remainder of this evaluation of the research progress made since the appearance of the paper of Handford et al. (1997) depicting how coaching of athletes at all levels of performance could advance, we will refer to two case studies as examples of the practical application of the conceptualization of ecological dynamics in modern professional sport, namely, Australian football (AF). Importantly, these case studies do not intend to offer a comprehensive empirical examination into the application of ecological dynamics. They provide readers with an initial “how to” perspective when attempting to integrate aspects of its theoretical propositions as discussed in the first part of this paper. We encourage other “practitioner-scientists” to continue to provide rich exemplars of its integration for performance preparation in the continued support of sport practitioners interested in how to apply its key concepts within their ecosystems.

## Part 2: Implications for the Work of Sport Practitioners

### Practitioners as Learning Environment Designers

This section offers two case studies of ongoing practice to exemplify how sporting practitioners have integrated the key components of ecological dynamics in their preparation for performance in elite AF. These examples should provide the reader with thought provocation, affording the opportunity to adapt the practice designs presented to suit the need of their ecosystem. Central to these examples, however, is the philosophical shift in how a sports practitioner perceives his role in preparation for performance, viewing themselves as learning environment designers rather than as prescribers of pre-programmed “optimal” movement solutions. It is hoped that these examples will demonstrate that viewing sporting practitioners as *sporting ecology designers* is not as provocative of a thought as perhaps initially perceived.

To instantiate these examples, we will (briefly) discuss the ontological shift that is required for sports practitioners evolving toward learning environment designers. For example, the integration of a “contemporary” approach to preparation for performance may challenge socio-cultural norms that have been engrained from generational traditions (Hodges and Baron, 1992). It is these socio-cultural norms that can subsequently constrain the emergence of new epistemologies (Hodges and Baron, 1992). Accordingly, practitioners are encouraged to theoretically anchor values or principles that shape their practice ecology, which may require a deep introspection of their role in preparation for performance. In these presented examples, sports practitioners were challenged to conceptualize themselves as the designer of an ecosystem that provides a rich landscape of affordances in the achievement of a task goal. In this broad ecosystem, the athletes were free to explore and inhabit certain regions of their landscape. The central tenet of the ecosystem, however, was predication on the notion of representative learning design (Pinder et al., 2011a). Put simply, the practice designs were to consist of a clear task goal predicated on informational constraints sampled from the competitive performance environment. The sporting practitioners subsequently built these informational sources into the ecosystem (“hands-on”) and then observed (“hands-off”) the emergent interactions that unfolded between the athlete and their environment. It was globally acknowledged that, through this interaction, athletes progressively attuned to the informational sources within their workspace, developing fine-grained relationships with their performance environment – described as developing *knowledge of* their environment rather than *knowledge about* their environment (Gibson, 1966; Araújo et al., 2009; Silva et al., 2013).

## Case Study 1: Informational Constraint Manipulation Shapes Ball Passing Interactions Between Players

### Introduction

Match-play within AF is contested between two teams of 18 (fielded) players, with the primary intention being to have outscored their opponents at the conclusion of the match. Thus, “match score” could be considered as a critical performance indicator (environmental constraint) that guides the players’ perceptions, intentions, and actions as they attempt to “manage a game” (i.e., either maintain or obtain the lead over the opposition team). The aim of this example was to demonstrate how the manipulation of key informational constraints (score) within a player’s performance environment can result in the emergence of self-organized behaviors as they exploit their environment to achieve a task goal. It is through careful practice design that players can develop a deeply integrated relationship with their performance environment, learning how to co-adapt to and direct the self-organization of their behaviors in response to emergent problems (thus, developing their *knowledge of* the AF performance environment).

### Methodology

#### Procedures

In this example, data were collected from seven match simulations performed during a preseason training phase within an elite Australian Football League (AFL) team. Each match simulation was performed in accordance with the regulation rulings imposed by the AFL (premier AF competition) and officiated by registered umpires. The two competing teams of 18 players were quasi-randomized across each of the seven match simulations, ensuring that neither team had a playing experience bias. Each match simulation was performed for a minimum of 20 min on separate training days across a 4-week period.

All match simulations were scored as in competitive AFL games (six points awarded for a “goal” and one point awarded for a “behind”). Prior to each match simulation, all players were instructed to play for their team to win. To enhance competitiveness, the players were informed of a penalty for the losing team. the players were informed that with ~3 min left to play within the match simulation, the scores would be manipulated to place one team in front by less than six points (a goal) irrespective of the current score. The scoreboard was visible to the players at all times throughout the match simulation. In addition to this information, the players in the separate teams were given 60 s prior to the start of each match simulation to postulate tactical actions that they perceived could exploit the constraint manipulation to achieve the task goal (winning the match simulation) pending the score (either being in front or behind by less than six points). The practitioners facilitated this process via the use of questioning (Chow et al., 2007), which directed the attention of the players to key affordances enabling possible solutions to the impending constraint manipulation (defined as the tactical problem). The important point to note here is that questioning from an ecological dynamics rationale does not involve the athletes providing verbalized reasoning and responses, which would emphasize the acquisition of what Gibson (1979) terms “*knowledge about*” the environment, framed by verbal descriptions. Rather, the aim of questioning is to direct the athletes’ attention to relevant affordances of the performance landscape so that they can respond to verbalized questions with “*knowledge of*” the performance environment (Gibson, 1966) expressed through actions, perceptions, and skilled intentionality (Button et al., 2020).

#### Data Collection

To observe the emergent responses to the informational constraint manipulation, a multidisciplinary approach was used, which consisted of a team of sports practitioners with expertise in different sub-disciplines of sport science. Each match simulation was filmed using three two-dimensional cameras positioned from behind the goals (frontal/posterior) and broadcast (sagittal) perspective. The augmented visual information was subsequently stacked such that each perspective was concurrently observable during video analysis, with the periods of the match simulations in which the informational constraint manipulation occurred being time-stamped to the vision.

To study emergent ball passing tendencies between players following the score manipulation, notational analysis was performed on all disposal types (kicks and handballs). In accordance with the constraint-led framework (Davids et al., 2008), performer, environmental, and task constraints were heuristically selected, being informed by prior work in AF (Woods et al., 2019a) and recommendations from an expert AF practitioner (defined by holding a senior coaching position within the AFL for more than 5 years) and skill acquisition specialist. These constraints are presented elsewhere (Woods et al., 2019a, b), but a brief description is provided here and in Table 1: possession time (task constraint) was defined as the time between the player first obtaining ball possession to the time of ball disposal. This was then split into two components – a possession in general play and a possession from a mark or stoppage (e.g., free kick) – and then into four temporal epochs. The environmental constraints were defined by the number of opposition players within a 3-m radius of the ball carrier at the point of ball disposal (carrier density) and the intended receiver of the passed ball at ball reception (receiver density). Performer constraints were defined relative to the locomotive characteristics of the player at the point of ball disposal – stationary (standing still or walking) or dynamic (jogging or running). The same performance analyst quantified these constraints across each of the seven match simulations using specific notational software (Sportscode version 11.2.18, Sportstec Inc., Warriewood, NSW, Australia).

**TABLE 1:** The constraint matrix used within this example.

Constraint class	Constraint	Description	Sub-category label
Task	Possession time (general play)	Time between a player obtaining and disposing of the ball while in general play (i.e., not from a “mark” or “free kick”)	0–1 s 1–2 s 2–3 s >3 s
	Possession time (stoppage)	Time between a player obtaining and disposing the ball from a stoppage in play (“mark” or “free kick”)	0–1 s 1–2 s 2–3 s >3 s
Environmental	Target density	Number of opposition players within a 3-m radius of the intended disposal target	Uncontested Even (e.g., 1 vs. 1) Superior (e.g., 2 vs. 1) Inferior (e.g., 1 vs. 2)
	Ball carrier density	Number of opposition players within a 3 m radius of the ball carrier at ball disposal	<1 opposition player (unpressured) 1 opposition player 2 opposition players 3 opposition players >3 opposition players
Individual	Disposal movement	Locomotive state at point of ball disposal	Stationary (e.g., walking) Dynamic (e.g., running)

*s, seconds; m, meters.*

#### Descriptive Analysis

All data were transformed to represent a percent of total disposals performed within each constraint class. The data were split into two categories: “pre-informational constraint manipulation” (i.e., before the score-imposed change) and “post-informational constraints manipulation” (i.e., after the score-imposed change), with descriptive statistics (mean) being calculated for each condition. A radar plot was used to visualize the distribution of the disposal percentage within each constraint category across both conditions (Woods et al., 2019a). This analytical approach was chosen as it afforded a relatively simple yet informative means of quantifying the emergent co-adaptability that ensued from the informational constraint manipulation.

### Results

**FIGURE 1:**
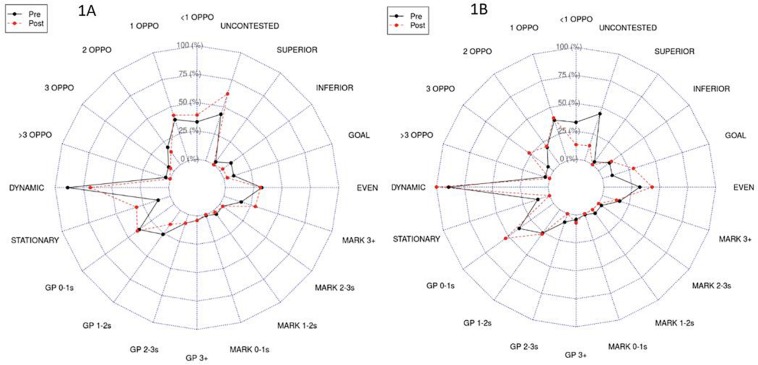
Radar plots demonstrating the mean differences between “pre” and “post” informational constraint manipulation for the team in front **(A)** and behind **(B)** following constraint manipulation.

As shown in Figure 1A, the team that was in front following the constraint manipulation possessed: (i) considerably fewer disposals performed within the 0–1 temporal epoch across both general play and stoppage constraint categories, (ii) a greater percent of total disposals performed from a stoppage in the > 3-s temporal epoch, (iii) a greater percent of total disposals to uncontested and superiorly numbered targets, and iv) fewer total disposals performed to inferiorly numbered targets relative to conditions prior to the score manipulation. Interestingly, this was in contrast to the team who was behind at the point of constraint manipulation (Figure 1B), exhibiting (i) fewer disposals to uncontested targets, (ii) fewer disposals performed with <1 opponent within a 3-m radius, (iii) greater disposals performed with >3 opponents within a 3-m radius, (iv) greater disposals in the 0–1 temporal epoch in general play, and (v) a greater percent of total disposals performed while running.

### Discussion

Collectively, the results of this case study indicated that the informational constraint manipulation (i.e., induced score change) led to the emergence of two distinct passing strategies utilized by players on either team: (i) one in which the players searched their workspaces for opportunities to slow their ball speed down and take lesser-risk disposal options when passing the ball to a teammate (1A) and (ii) another in which players searched their workspaces for opportunities to speed up their ball movement at the expense of seeming to take riskier disposal options when passing the ball to a teammate (1B). Specifically, the strategy demonstrated in 1A appeared to reflect a team who was “resting with the ball” in a somewhat conservative attempt to preserve their lead following the informational constraint manipulation. Conversely, the strategy demonstrated in 1B appeared to be one in which the players “threw caution to the wind” in an attempt to optimize their perceived likelihood to score. To further these insights, practitioners could consider the use of more advanced machine learning techniques such as rule association (Browne et al., 2019). Such an approach extends the descriptive analysis described here through the appreciation of the interaction between nested task constraints, offering greater insight into the combination of constraints that are likely to shape the disposal characteristics in response to an emergent “tactical problem” experienced within the competition.

Beyond these nuanced findings, this example demonstrates the utility of a practice design conceptualized through ecological dynamics. Specifically, this practice design afforded opportunities for players to build deeper relationships with their competitive environment, exhibiting *skilled intentionality* (Rietveld and Kiverstein, 2014) through the collective co-adaptability shown in their passing strategy relative to the informational constraint manipulation. This observation echoes our sentiment discussed earlier in this commentary that “behaviors” do not occur in a vacuum but, rather, through the ecological dynamics lens; “skilled behaviors” are functionally adaptable performance solutions that arise from the continuous interactions that an organism shares with their environment (referred to as skill adaptability; Araújo and Davids, 2011).

## Case Study 2: Inviting Deceptive Behavior Through Informational Constraint Manipulation

### Introduction

An important design feature of practice tasks in AF is the presentation of affordances where time and space are manipulated to channel successful ball disposal actions between teammates (Robertson et al., 2016). Thus, providing opportunities for players to explore behaviors that could successfully deceive their opponents in the search for time and space should be included within preparation for performance models. The intention of this second case study is to offer the reader insights into how sports practitioners may design a practice activity that solicits deceptive behaviors. Specifically, this example presents a practice task that intends to provide a rich landscape that promotes the exploration of deceptive behavior in AF.

### Methodology

#### Procedures

**FIGURE 2:**
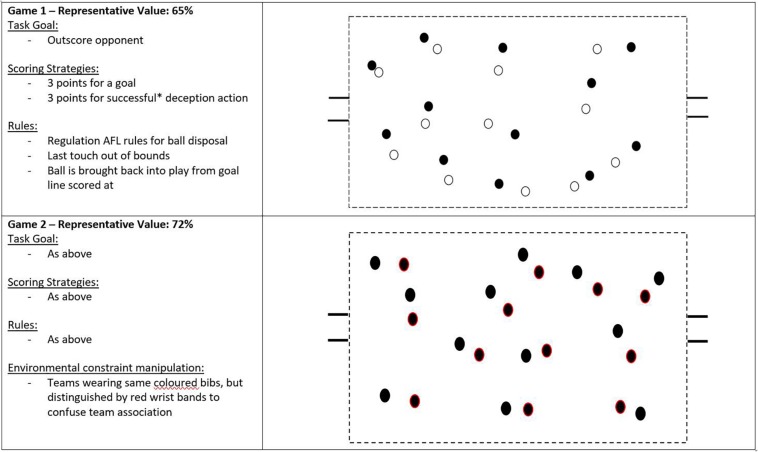
Practice design for two activities that are designed to offer deceptive action opportunities – note the representative values that have been calculated using the methodology described by Farrow and Robertson (2017) and applied by Woods et al. (2019b); *A successful deceptive action was defined as one that coerced an opponent into a movement pattern that was exploited. The dots denote players.

The same population as described in the first case study was used here. The two practice tasks designed to invite deceptive behaviors are presented in Figure 2. Both practice tasks were performed once (14 min in duration) during a pre-season phase of performance preparation. First, the subtle scoring system used within both games is worth noting (Figure 2). Given that the task goal of both games was to outscore their opposition, the points awarded for a successful deceptive action immediately led to the emergence of a landscape where deceptive actions were afforded and solicited. Further, it is important to note the environmental constraint manipulation in the second game. Specifically, team association was convoluted through the use of the same colored bibs for both teams, with the players being distinguishable only through the use of a colored wristband. This constraints manipulation methodology was used to encourage the players to explore unique ways to achieve the task goal relative to the first game. Additionally, the utility of such a constraint manipulation was informed from prior work describing the development of expertise in soccer, where team convolution was discussed as a common strategy that promoted scanning and deceptive behaviors (Uehara et al., 2018). To direct the attention to key informational sources of the task for the exploration of deceptive behaviors, the players discussed for 60 s prior to the start of each game about task configurations and possible behaviors that they perceived could be performed to deceive their opponent. As was done in the first case study, the practitioners facilitated this process via the use of questioning to elicit knowledge of the performance environment (Chow et al., 2007).

#### Data Collection

As was done in the first example, a multidisciplinary approach was used to observe the emergent deceptive behaviors. Both games were filmed using three two-dimensional cameras positioned from a behind the goals (frontal/posterior) and broadcast (sagittal) perspective. The augmented visual information was subsequently stacked such that each perspective was concurrently observable during video analysis. To quantify emergent deceptive actions, notational analysis was used (Sportscode version 11.2.18, Sportstec Inc., Warriewood, NSW, Australia). Specifically, “successful deceptions” were coded and categorized into one of five categories, with a description of each category being provided in Table 2. These deception categories were chosen and defined in accordance with the sports practitioner’s experiential knowledge.

**TABLE 2:** Deception categories and subsequent descriptions.

Deception category	Description
Faked disposal	An action of ball disposal that led an opponent to move in a different direction to where the ball was subsequently disposed
Creative disposal	An “unconventional” means of ball disposal that successfully reached its intended target (e.g., handballing between the legs of one’s direct opponent)
Calling for the ball in defense	An act of calling for and receiving the ball from an opponent while in defense
Teammate blocking an opponent	An act of physically blocking an opponent from a teammate who is in possession of the ball
Other	Any emergent deceptive action that was undefined in the above

#### Descriptive Analysis

All data were transformed to represent a percent of total deceptive behaviors performed within each category, enabling a simple comparison relative to the constraint manipulation. Following this, a bar graph was used to visualize the distribution of the deceptive behaviors across both games.

### Results

**FIGURE 3:**
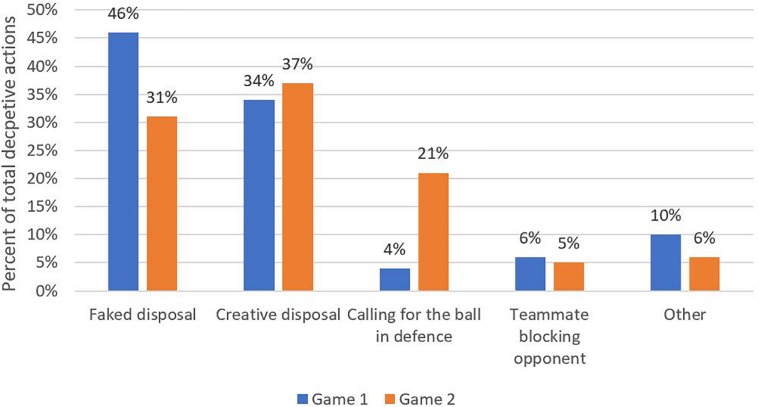
The percentage of total deceptive actions observed in both games 1 and 2.

The most commonly observed deceptive behavior in the first game was the “faked disposal,” followed by the “creative disposal” (Figure 3). This observation indicates that the most common solicitations for deceptive actions afforded in the first game involved movement adaptability relative to an opponent for the player in possession of the ball. Interestingly, however, while both “faked disposals” and “creative disposals” still remained as primary deceptive behaviors in the second game, “calling for the ball in defense” emerged as a prominent strategy for deceptive actions relative to the first game (Figure 3). It was likely that the augmentation for this was the additional environmental constraint manipulation that convoluted team association. Specifically, the players appeared to exploit this environmental constraint while in defense by hiding their wristband and calling for the ball from their opponent. This indicates that the additional environmental constraint manipulation invited more exploratory deceptive actions for the players when they were not in possession of the ball, relative to the first game.

### Discussion

Collectively, this example demonstrates the utility of practice design framed through ecological dynamics where the sports practitioner designs a rich landscape that affords opportunities for a specific action to emerge. In this rich affordance landscape, the players were free to accept or reject invitations for action. From this perspective, the use of constraint manipulations directed, or guided, the players’ attention toward the exploration and the exploitation of performance invitations (affordances) within their environment relative to their current action capabilities. For example, given that a specific performance solution was not prescribed in this practice task, the players were free to undertake any form of deceptive manoeuver that they felt could exploit their opponent based on the constraints designed in (e.g., score system and team convolution). It is presumed that through this design, the players would progressively learn to couple their movements to the opportunities presented and detected within their environment, progressively “acquiring” a deeper *knowledge of* (Gibson, 1966) their environment through the development of their perception–action coupling. Thus, in this case study, “hands-on coaching” occurred through the practice design rather than through the provision of prescriptive instructions of how to deceive an immediate opponent (i.e., how to perform a “football action”).

This more ecological perspective of practice task design draws a stark contrast to the more traditional, linear approach. Specifically, framed through a more traditional perspective, it is likely that the target football action (in this case, a deceptive movement) would have been practised in a de-contextualized manner in isolated, unopposed practice based around the reproduction of a putative gold-standard movement template. Contrastingly, the practice task design framed through ecological dynamics offers the practitioners with a different perspective of skill “acquisition,” being the development or “acquisition” of the performers’ functionally adaptable relationship to their performance environment, which can be fostered through targeted and careful constraint manipulation, not the repetition of an uncoupled and physically reproducible “technique.”

## Concluding Remarks

As poignantly highlighted by McChrystal et al. (2015) in the opening quotation, gardeners do not actually grow a plant; rather, they facilitate an environment to which vegetation adapts and in which plant growth emerges. This commentary and set of case studies sought to foster reflection in readers on the alignment of key ideas in this framework and the fundamentals of preparation for performance models in sport. Pertinently, this practice ecology was originally discussed over two decades ago by Davids et al. (1994) and Handford et al. (1997), who proposed the notion of an ecological approach to skill “acquisition.” In their propositions, sports practitioners were urged to appreciate the complex and deeply integrated reciprocity of the organism (performer), task, and environment subsystems, which signaled a change in how their role was conceptualized in preparation for sport performance. Over two decades later, we have seen the continued evolution of this rationale through the contemporary theoretical lens of ecological dynamics. Through this theoretical rationale, sports practitioners are now afforded a guiding framework that fosters many areas of sport science, such as skill “acquisition,” practice and training design, performance analysis and preparation, and talent development. We proposed how the framework of ecological dynamics could support the integrated work of an extensive group of sport practitioners in a DoM in sports organizations dedicated to athlete development and preparation for performance.

Indeed this is an exciting era for sports practitioners and applied scientists interested in augmenting athlete performance. We now find ourselves on the cusp of the next “frontier” of ecological dynamics, one which sees the offering of rich exemplars as to how teams of sports practitioners have successfully integrated its propositions into preparation for performance models. To continue to aid this progress, we propose that sports practitioners should conceptualize themselves through a different light, one which sees them appreciating the non-linearities of human behavior, and design ecosystems that have the athlete–environment interaction at its core. It is perhaps through this conceptualization that sporting practitioners may actually see that viewing themselves as *sport ecology designers* is not as farfetched as initially thought.

## Ethics Statement

The studies involving human participants were reviewed and approved by James Cook University Human Ethics Committee. The organization provided their written informed consent to participate in this study.

## Author Contributions

CW and KD conceptualized the paper, while CW and IM established the case studies. CW, KD, SR, MR, and DA each contributed to and drafted the first section of the paper, while CW, KD, SR, DA, and IM wrote and drafted the second section of the paper. All authors contributed to the manuscript revisions based on a reviewer’s commentary.

## Conflict of Interest

The authors declare that the research was conducted in the absence of any commercial or financial relationships that could be construed as a potential conflict of interest.
